# Crashworthiness Analysis of Bio-Inspired Multi-Cell Concave Tubes

**DOI:** 10.3390/biomimetics11020120

**Published:** 2026-02-06

**Authors:** Xiaolin Deng, Jinjin Huang, Jialiang Xie

**Affiliations:** 1 School of Mechanical and Resource Engineering, Wuzhou University, Wuzhou 543002, China; 2 School of Mechanical and Electrical Engineering, Guilin University of Electronic Technology, Guilin 541004, China; huangjinjin0104@163.com (J.H.); xiejialiang_2020@163.com (J.X.)

**Keywords:** crashworthiness, thin-walled structures, bio-inspired, multi-cell concave tube, hierarchical

## Abstract

This study presents a novel bio-inspired multi-cell concave tube (BMCT) inspired by the biomimicry of horse tail grass plants. Following the simulation validation, a comprehensive investigation into the crashworthiness of this structure under axial impact was conducted. Concurrently, both experimental and theoretical analyses were employed to substantiate the reliability of the simulation data. Comparative results concerning crashworthiness indicate that, relative to other structures, the BMCT maintains a relatively constant initial peak force while simultaneously enhancing energy absorption capacity at equivalent mass. Specifically, when compared to corresponding hierarchical multi-cell tubes with the same number of cells, the BMCT exhibits a 41.04% increase in crush force efficiency (CFE) while preserving a relatively unchanged initial peak crushing force (IPCF). Additionally, variations in hierarchical levels yield a 21.22% increase in CFE at the same mass.

## 1. Introduction

Over millions of years of evolution in nature, living organisms have developed highly optimized structural and functional characteristics, serving as a profound source of inspiration for contemporary engineering design and materials science [1,2,3,4]. Among the diverse organisms found in nature, plants exhibit remarkable advantages in mechanical properties, energy absorption, and adaptability due to their unique structural features, such as honeyscomb tissue, fractal branches, and gradient porous structures. For example, bamboo demonstrates exceptional bending and impact resistance attributable to its segmented hollow structure and fiber gradient distribution [5,6]; similarly, various multicellular structures in horsetail plants can withstand loads from wind and their own weight [7,8]. Consequently, the structural characteristics of these organisms have laid the groundwork for the development of biomimetic structures. The efficiency of these biological architectures has inspired researchers to implement biomimetic strategies in engineering materials and structural design, aiming to enhance the mechanical properties, energy absorption efficiency, and multifunctionality of synthetic materials [9,10].

Bionic multicellular tube structures (including honeycomb tubes [11,12], sandwich tubes [13,14], bionic composite laminated plates [15], corrugated tubes [16,17,18], multicellular tubes [9,19,20], and bionic layered tubes [21]) hold significant potential for applications in aerospace, automotive crash protection, and building fortification due to their lightweight nature, high specific strength, and outstanding energy absorption capabilities [22,23,24,25]. Nonetheless, traditional multicellular tubes frequently experience local failure due to stress concentration when subjected to dynamic impact or compressive loads, thereby compromising overall energy absorption efficiency. To address this challenge, researchers have increasingly begun to explore bionic multicellular structures by emulating the microscopic and macroscopic structural features of plants and animals (such as graded pores, hierarchical arrangements, and concave geometries) to optimize mechanical properties [26]. Research on bionic plant structures predominantly focuses on several aspects: graded porous structures, exemplified by the stems of lotus leaves and the graded pore distribution in wood, which exhibit excellent performance in energy absorption and stress distribution optimization [9]; hierarchical arrangements, as seen in the fiber-reinforced structures of bamboo nodes, which achieve high toughness and impact resistance through multi-scale arrangements [10,27]; and concave geometric configurations, where the tubular structures of certain plant stems possess concave polygonal cross-sections [26,28,29], effectively enhancing bending and compressive resistance [30].

In engineering applications, the design of biomimetic multicellular tubes has made notable progress. For instance, hexagonal multicellular tubes inspired by bee hives demonstrate superior specific stiffness and energy absorption capacity [20,31,32,33]. Furthermore, biomimetic tubes modeled after the spiral structures of conch shells exhibit a progressive failure mode in impact resistance, effectively avoiding the abrupt failure characteristic of traditional structures [21,34]. However, current biomimetic research regarding the concave structures found within plants, such as the concave polygonal cross-sections of certain herbaceous plant stems, remains relatively limited. These unique geometric features may offer novel insights for optimizing the mechanical properties of multicellular tubes.

Despite the advancements made in the study of biomimetic multicellular tubes, several challenges persist: insufficient structural optimization, as traditional biomimetic designs often concentrate on a single structural feature (e.g., honeycomb or corrugation), while the advantages of plant structures frequently arise from the synergistic interaction of multiple features (e.g., gradient, concavity, and hierarchy); limitations in manufacturing processes, as the precise fabrication of complex biomimetic structures (such as concave multicellular tubes) necessitates stringent requirements for additive manufacturing (3D printing) or precision casting technologies; and an unclear understanding of the dynamic mechanical mechanisms, as the energy absorption mechanisms of plant biomimetic structures under high-speed impact warrant further investigation, particularly concerning the influence of the concave configuration on plastic deformation behavior. In the past, bionic tubes or concave tubes were usually coupled with other features separately, and the combination of the two was relatively rare. To address these issues, this study proposes a bio-inspired multi-cell concave tube (BMCT), featuring innovations such as geometric configuration optimization by mimicking the concave polygonal cross-section of plant stems and integrating gradient cell distribution to enhance energy absorption efficiency; mechanical performance enhancement through finite element simulation and experimental verification to explore the response mechanisms of the concave structure under compression, bending, and impact loads; and multifaceted application potential by assessing the applicability of this structure in fields such as automotive crash protection and aerospace shock absorbers.

The horsehair structure, with its unique hollow and segmented design, transforms solid materials into hollow tubes through material mechanics. This design dramatically increases the cross-sectional moment of inertia while using minimal material, making it more effective at resisting bending deformation. This principle is applied in bicycle frames and aircraft wing skeletons. The stem of the horsehair grass itself serves as a natural lightweight yet high-strength tube. Given these advantages, we decided to design it as a thin-walled structure. This study aims to systematically investigate the mechanical properties and energy absorption mechanisms of concave-biased multicellular tubes through bionic design, numerical simulation, experimental testing, and theoretical analysis. The structure of the paper is organized as follows: Section 2 elucidates the bionic design principles and the geometric modeling methods for concave multicellular tubes; Section 3 establishes a finite element model and conducts quasi-static compression and dynamic impact experiments using 3D-printed samples; Section 4 employs finite element analysis (FEA) to simulate the static and dynamic mechanical behavior and compares it with other structures; Section 5 presents a theoretical analysis of the structural energy absorption; and Section 6 summarizes the research conclusions and outlines future work. Through this study, we anticipate providing innovative biomimetic concepts for the design of high-performance energy absorption structures and contributing to the further application of plant bionics in the field of engineering materials.

## 2. Materials and Methods

### 2.1. Structural Design

**Figure 1:**
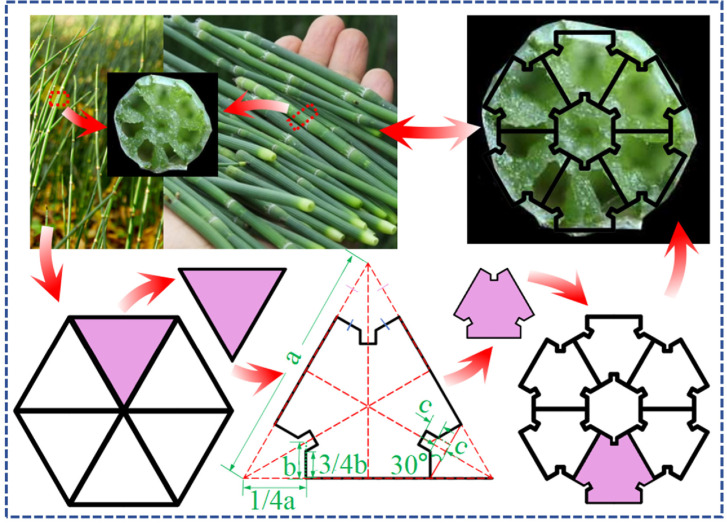
Structural design.

In nature, numerous plant structures exhibit exceptional mechanical properties, exemplified by wheat [35] and the structure of horse tail [36]. As illustrated in Figure 1, the distinctive hollow and segmented architecture of these structures, when analyzed from a materials mechanics perspective, reveals that the transformation of a solid material into a hollow tubular configuration can significantly enhance the moment of inertia of the cross-section while minimizing material usage. This transformation allows for more effective resistance to bending deformation. The principles derived from such structures are employed in the design of bicycle frames and the frameworks of aircraft wings. The stem of horse tail grass serves as a naturally lightweight and high-strength tubular structure. Drawing inspiration from its cross-sectional design, we have developed a multi-cell structure composed of multiple concave sub-units.

**Figure 2:**
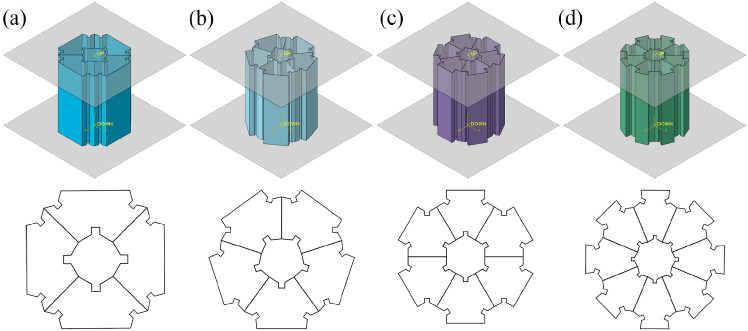
Bio-inspired multi-cell concave tube (BMCT): (**a**) square multi-cell concave tube (SMCT), (**b**) pentagonal multi-cell concave tube (PMCT), (**c**) hexagonal multi-cell concave tube (HMCT), (**d**) octagonal multi-cell concave tube (OMCT).

The article considers the triangle as the foundational sub-unit of the structure, subsequently partitioning the polygon into a corresponding number of triangles based on its sides. The relationship of size correspondence between the concave sub-units and the initial triangular sub-units is illustrated in Figure 1. By systematically reducing the number of sides of the polygon from an octagon to a hexagon, then to a pentagon, and finally to a quadrilateral, four distinct groups of biomimetic concave multi-cell tubes (bio-inspired multi-cell concave tube, BMCT) are identified within this study. These include the square multi-cell concave tube (SMCT), pentagonal multi-cell concave tube (PMCT), hexagonal multi-cell concave tube (HMCT), and octagonal multi-cell concave tube (OMCT). Figure 2 presents the simulation models and cross-sectional sketches of the corresponding structures.

### 2.2. Material Property

**Figure 3:**
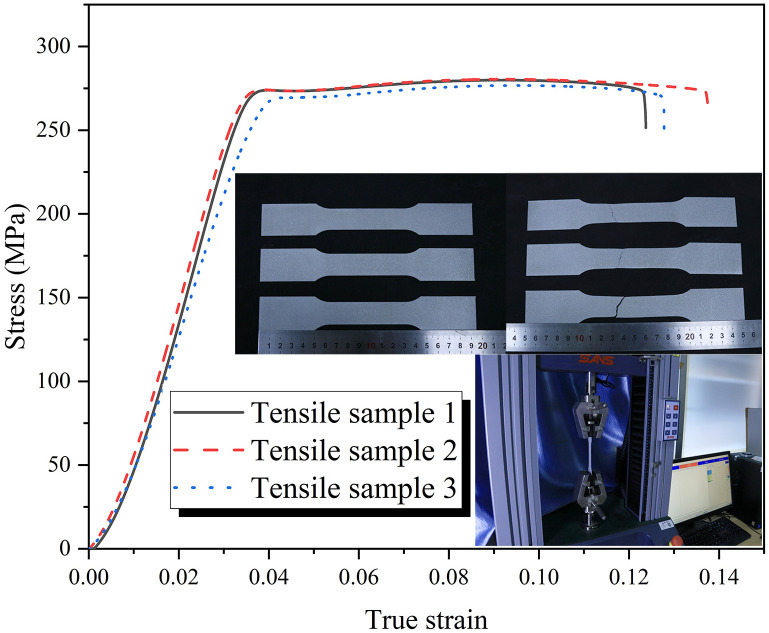
Engineering stress-strain curve of AA6061O [37].

The material utilized in this study is aluminum alloy, AA6061-O [37]. The engineering stress-strain curve for this material is presented in Figure 3. The density of the material is provided as $\rho =2.7\times 10^{3}\;kg/m^{3}$, and the mechanical properties are characterized by Young’s modulus, E=68 GPa, and Poisson’s ratio, ν=0.33. Initial yield stress is expressed as $\sigma_{y}=71\;MPa$, and ultimate stress is expressed as $\sigma_{u}=130.7\;MPa$.

### 2.3. Crashworthiness Indexes

Various indicators [36,37] exist for assessing the energy absorption capacity of different structures, as illustrated in Table 1, where m is the mass, d is the impact distance, and IPCF is the initial peak crushing force.

## 3. Digital Model

### 3.1. Finite Element Model Establishment

**Figure 4:**
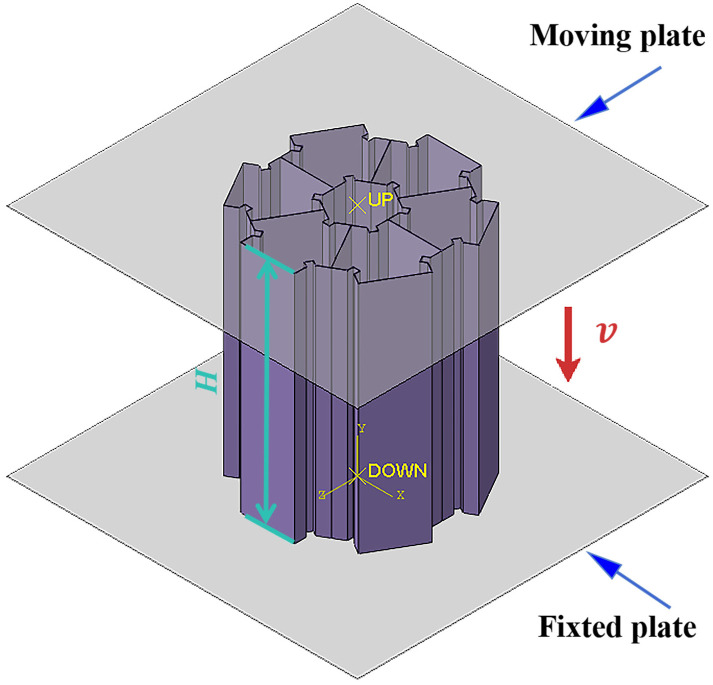
Finite element model.

This paper carries out numerical simulation using Abaqus, and the finite element model is shown in Figure 4. In the numerical simulation, the pipe fittings are placed between two rigid plates. We apply a completely fixed constraint to the lower rigid plate and bind it to the pipe fittings. The upper rigid plate is subjected to a vertical downward impact at a constant speed of 10 m/s [38], with the impact displacement set at 80% of the pipe length (80 mm). The simulation is carried out using a four-node reduced-integration shell element. To ensure convergence, five integration points are used along the thickness direction. The model includes a self-applied general contact algorithm, with the friction coefficient set to 0.2 [13].

### 3.2. Mesh Test

**Figure 5:**
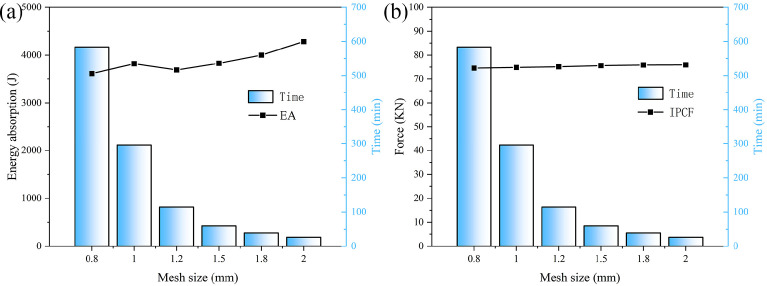
Mesh size analysis results: (**a**) energy absorption curve, (**b**) force-displacement curve.

The mesh size of the finite element model exerts a significant influence on the results obtained. Consequently, a convergence analysis encompassing six distinct mesh sizes—specifically 0.8 mm, 1.0 mm, 1.2 mm, 1.5 mm, 1.8 mm, and 2.0 mm—was performed in this study. The HMCT model was utilized with a wall thickness of t = 1 mm. The results are illustrated in Figure 5a,b, with detailed data presented in Table 2. As indicated in Table 2, the mesh size markedly affects energy absorption; however, it has a negligible impact on the initial peak force. The difference in energy absorption between a mesh size of 1.0 mm and 0.8 mm is merely 0.89%. In contrast, when comparing the energy absorbed by the structure at a mesh size of 2.0 mm to that at 0.8 mm, the difference is as substantial as 18.51%. In the same context, the difference for the IPCF is only 2.65%. This clearly illustrates that variations in mesh size have minimal impact on IPCF. Furthermore, Figure 5 demonstrates that when the mesh size is either 0.8 mm or 1.0 mm, the required computational time is significantly greater than for the other mesh sizes. Taking into account the potential errors and computational costs, a mesh size of 1.2 mm was ultimately selected for subsequent analyses.

### 3.3. Model Validation

To ensure the validity of the finite element model, it is essential to juxtapose its outcomes with those obtained from quasi-static compression experiments. The finite element simulation is a widely recognized methodology within the engineering domain. When appropriately calibrated, it can yield high-precision results, thereby not only reducing costs but also ensuring that the outcomes align with the practical requirements of engineering applications. The dimensions of the structure are consistent with those of the prior structural design; specifically, the external regular hexagon has a side length of 48.188 mm, a height of 100 mm, and a wall thickness of 1.0 mm, with the material being AA6061-O. The metal model was made by a commissioned factory. The verification was conducted through a quasi-static compression experiment. The model of the universal testing machine was SANS CMT5205, with a loading speed of 2 mm/min and a compression displacement of 80 mm. To ensure the validity of the numerical model, the geometric dimensions and loading parameters in the finite element analysis were all consistent with the experiment.

**Figure 6:**
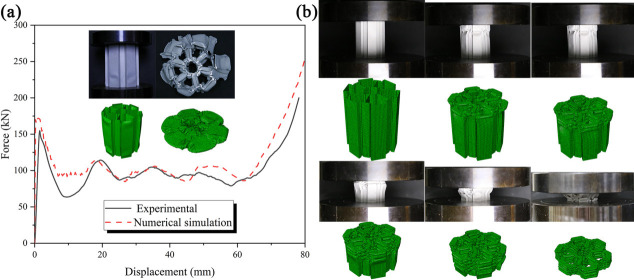
Comparison between experiment and numerical simulation. (**a**) t_m_ = 1.0 mm;(**b**) t_m_ = 0.5 mm.

Figure 6a presents the force-displacement curves from both the experiment and simulation. The experimental findings reveal that the deformation mode observed in the tests closely mirrors that of the simulation. Given that densification was achieved at approximately 78 mm, a comparative analysis of energy absorption and average force at this compression level was conducted. From Figure 6a, it can be deduced that the absorbed energy prior to densification in the experimental and simulation contexts was 7728.912 J and 8576.269 J, respectively, indicating a mere 9.88% discrepancy. During the experiment, the energy absorption was marginally lower than that projected by the simulation due to the ultimate rupture of the structure. Additionally, as illustrated in Figure 6a, the disparity in average force between the experimental and simulation results is minimal, with trends exhibiting significant similarity.

The observed similarities in deformation between the experimental and simulated results suggest that factors such as model grid size, processing accuracy, and experimental apparatus are the primary contributors to any discrepancies. In conclusion, we assert that the finite element model possesses adequate accuracy for subsequent simulation studies. Notably, numerical simulation has found extensive application in the crashworthiness analysis of thin-walled structures [39,40]. Through comprehensive analysis, it has been confirmed that the accuracy of the finite element model meets the requisite standards. The settings, boundary conditions, and loading conditions for the subsequent simulation will remain consistent with those used in the initial verification. Consequently, we contend that the forthcoming simulation experiment is of substantial significance. Simultaneously, we modified the wall thickness to 0.5 mm and conducted a second series of experiments, yielding the deformation mode comparisons depicted in Figure 6b. This indicates that wall thickness exerts a discernible influence on the deformation mode of the model during compression.

## 4. Crashworthiness Analysis

### 4.1. Comparison of the Same Quality

**Figure 7:**
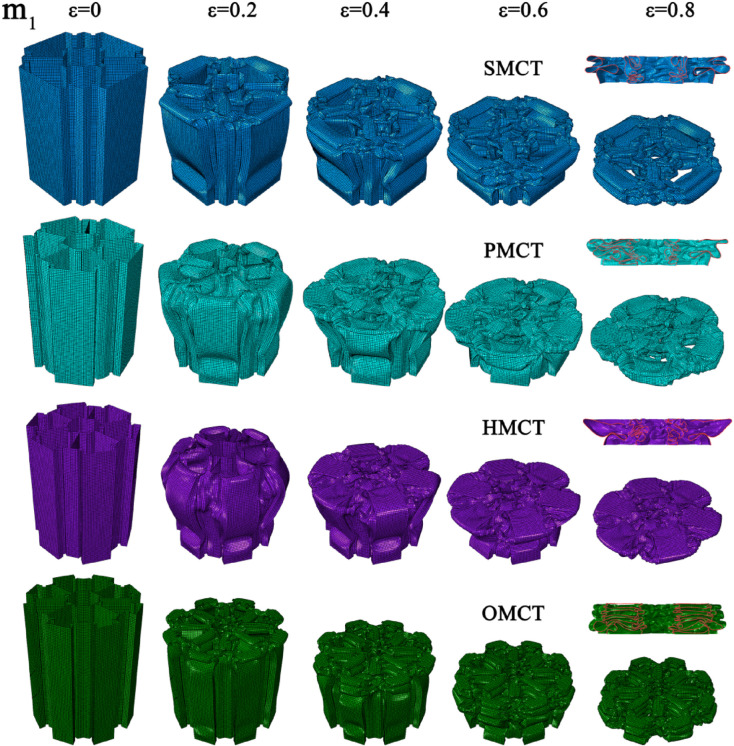
Deformation mode of the BMCT with t_m_ = 0.8 mm.

**Figure 8:**
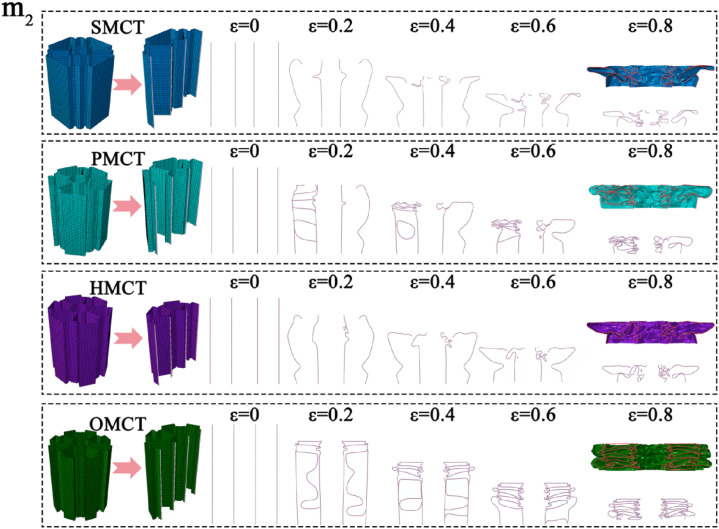
Deformation mode of the BMCT with t_m_ = 1.0 mm.

**Figure 9:**
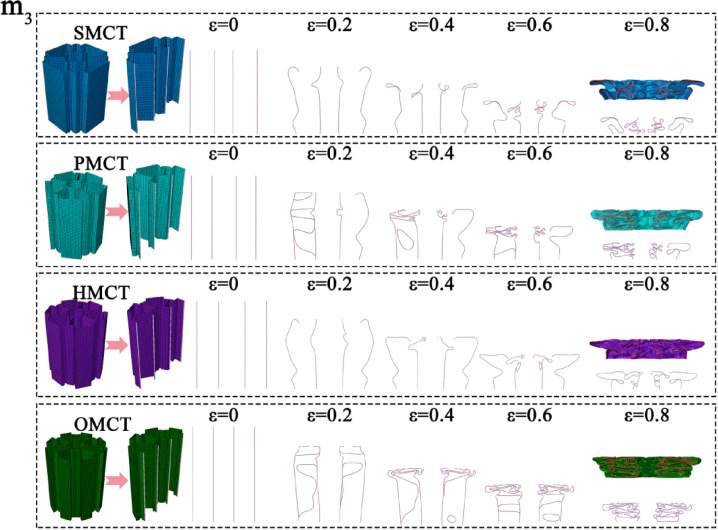
Deformation mode of the BMCT with t_m_ = 1.2 mm.

**Figure 10:**
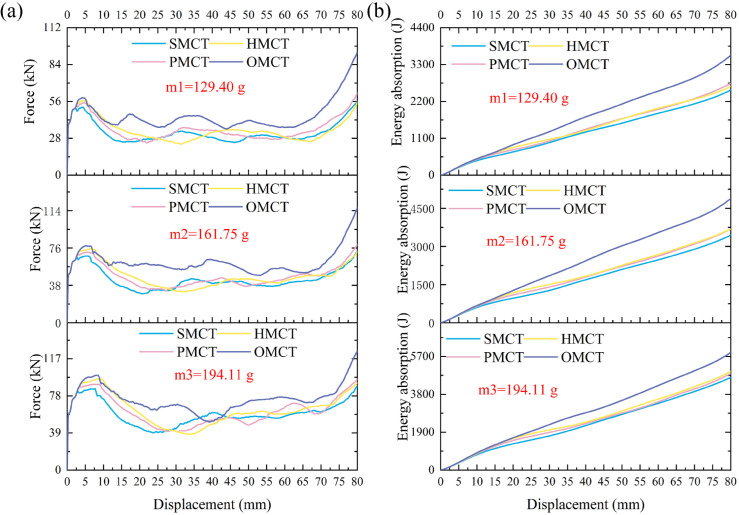
Force-displacement curve and energy absorption curve of different t_m_. (**a**) Force–displacement curve and (**b**) energy absorption curve of different t_m_.

**Figure 11:**
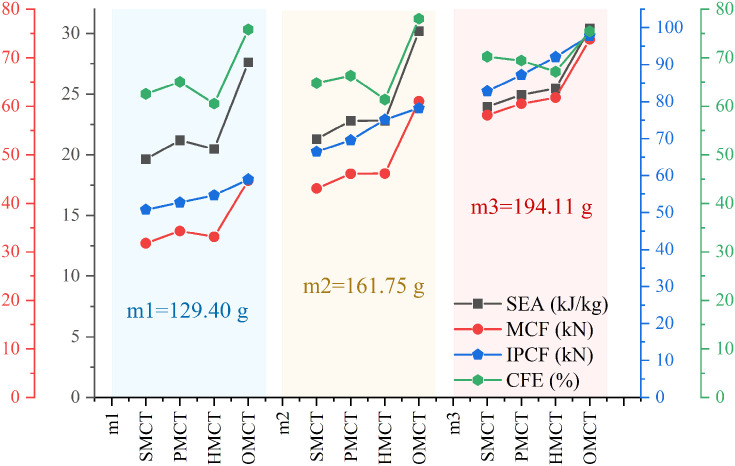
Crashworthiness data comparison of different tm.

In this section, we establish three reference groups, each containing four structures of SMCT, PMCT, HMCT, and OMCT with the same mass. The wall thickness of each structure is different, with the wall thickness of HMCT serving as the benchmark. The wall thicknesses of HMCT in each group are t = 0.8 mm (Group 1), t = 1.0 mm (Group 2), and t = 1.2 mm (Group 3). Under the same mass condition, each group studies the impact of different levels on the structural crashworthiness under axial impact. Comparisons between different groups reveal the influence of wall thickness on the structural crashworthiness. The crashworthiness data acquired from finite element numerical simulations conducted under axial impact for each group are presented in Table 3. Based on the simulation outcomes, comparative diagrams illustrating the deformation modes at equivalent wall thickness, as well as energy absorption curves, force-displacement curves, and crashworthiness index comparisons under the same wall thickness, are depicted in Figure 7, Figure 8, Figure 9, Figure 10 and Figure 11, where ε denotes the strain of the compressed structure.

The corresponding deformation modes are illustrated in Figure 7, Figure 8 and Figure 9. The analysis indicates that under conditions of uniform wall thickness, while disregarding the effects of IPCF, an increase in the number of layers—with the BMCT wall thickness held constant—results in the formation of distinct structural units due to the connecting rib plates introduced in each layer. The augmentation in the number of rib plates directly enhances the structural integrity and energy absorption capabilities of the folded angle unit. During impact deformation, shorter fold wavelengths are more readily formed, thereby leading to a notable increase in the number of folds. This phenomenon culminates in a more uniform progressive folding mode, thereby enhancing the structural resistance to impact. However, the central symmetry inherent in the HMCT sub-unit structure results in the loss of multiple Y-shaped configurations, which possess greater energy absorption capacity (a detailed discussion is available in Chapter 5, Theoretical Analysis). Consequently, this leads to a marked reduction in certain crashworthiness indicators due to suboptimal deformation conditions. As indicated in Table 3, an increase in wall thickness corresponds proportionally to variations in the structural crashworthiness of BMCT, wherein MCF, SEA, and CFE exhibit increases. Notably, as the cross-section becomes more complex, IPCF also experiences a slight increase.

Figure 10 and Figure 11 illustrate the force-displacement and energy absorption curves for each group of structures. As depicted in Figure 10, within each group of BMCT structures—excluding the HMCT structure—there is a discernible increase in both force and energy absorption with rising hierarchy levels. Furthermore, the variations in wall thickness significantly influence the force-displacement and energy absorption characteristics of the structures. Figure 11 demonstrates that, apart from the HMCT structure, within the same group and wall thickness, the crashworthiness of the structures improves as the hierarchy ascends. Table 3 reveals that the specific energy absorption (SEA) exhibits a maximum increase of 41.78%, while the crush force efficiency (CFE) shows an enhancement of 21.22%.

### 4.2. Compared with Other Structures

**Figure 12:**
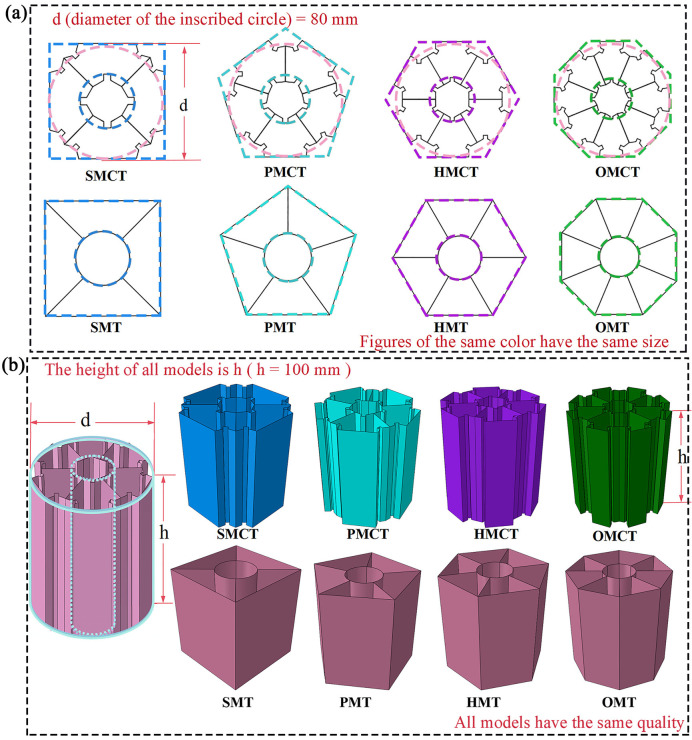
The structural model for comparison and its parameters: (**a**) Plan view sketch, (**b**) Simulation model.

In this section, we conducted a comparative analysis of multi-cell structures that possess an identical number of cells, congruent circumscribed polygons, and equivalent circumscribed circles concerning their internal configurations as delineated in this article. Figure 12 illustrates the cross-sectional sketches and simulation models of each structure under comparison, as well as the dimensional correlations among them.

**Figure 13:**
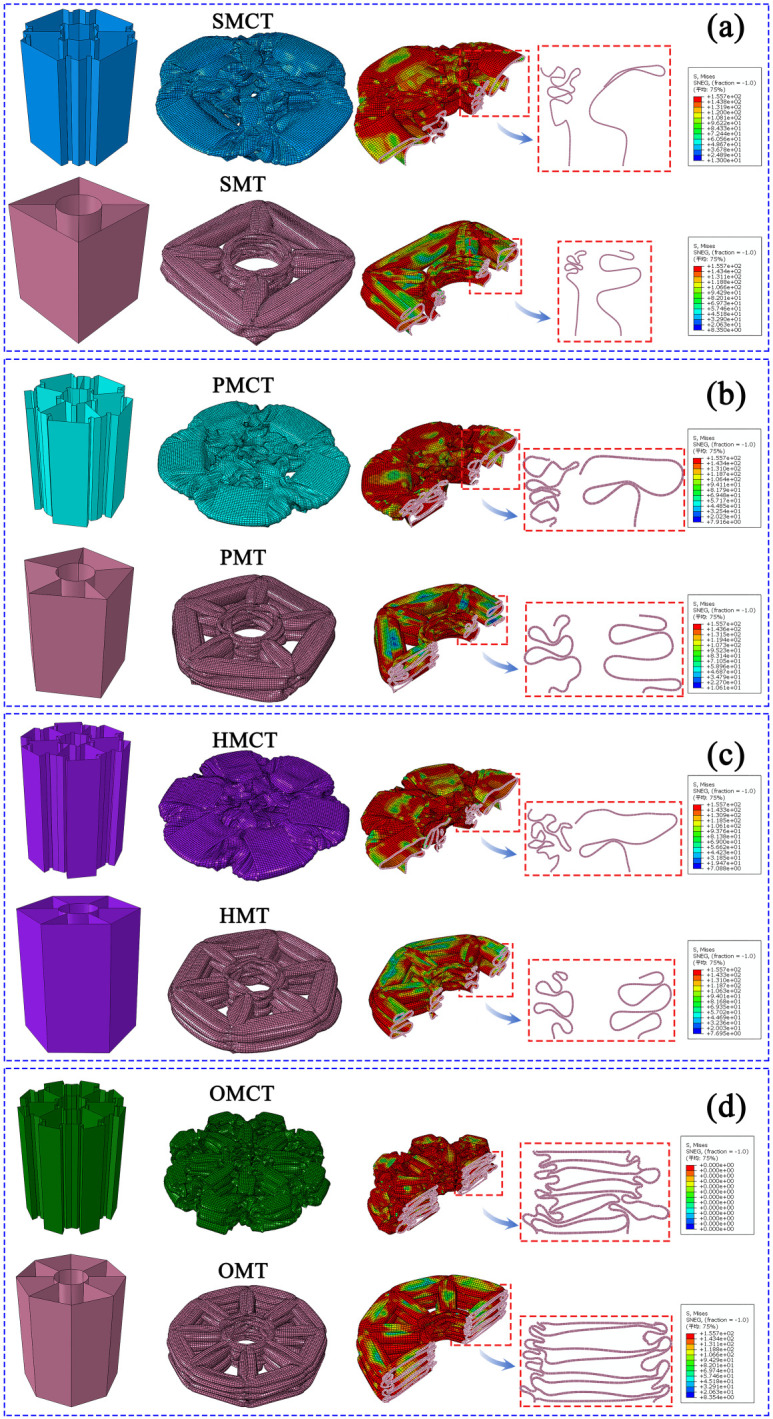
Comparison of deformation patterns of BMCT and BMT. (**a**)SMCT and SMT; (**b**)PMCT and PMT; (**c**) HMCT and HMT; (**d**) OMCT and OMT.

**Figure 14:**
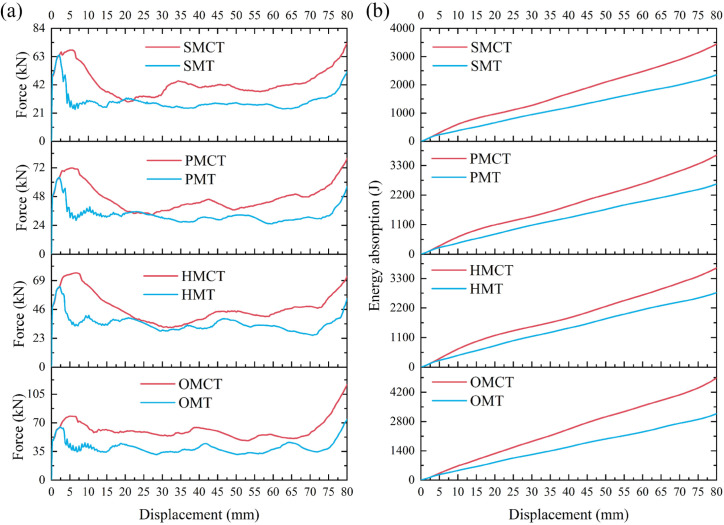
Force-displacement curve and energy absorption curve. (**a**) Force–displacement curve and (**b**) energy absorption curve.

**Figure 15:**
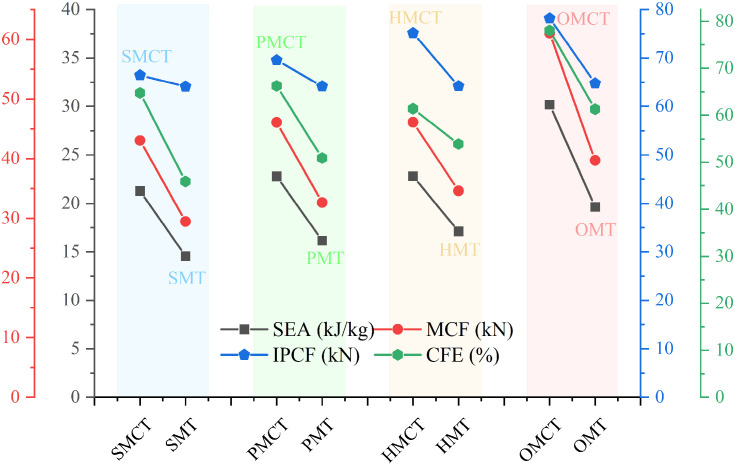
Crashworthiness data comparison.

**Figure 16:**
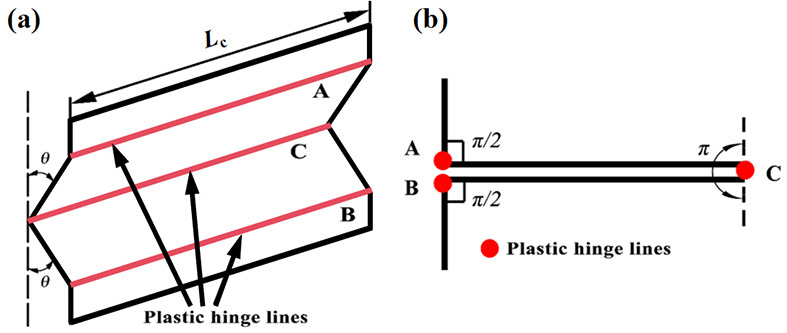
Flange deformation: (**a**) plastic hinge line, (**b**) angle of rotation [19].

This section establishes the mass of m = 161.7542 g for OMCT, with a wall thickness of 1.0 mm, as the standard for comparison. Under the condition that the masses of all structures being analyzed are equivalent, a comparison is made between BMCT (including SMCT, PMCT, HMCT, and OMCT) and BMT (including SMT, PMT, HMT, and OMT). The crashworthiness data for each group of structures, obtained through finite element numerical simulations under axial impact, are presented in Table 4. Based on the simulation outcomes, deformation mode diagrams and energy absorption curves corresponding to the same mass are illustrated in Figure 13 and Figure 14. Additionally, the force-displacement curves are depicted in Figure 15, along with comparative diagrams of the crashworthiness index at equal wall thickness, as shown in Figure 16.

The corresponding deformation modes and cross-sections are illustrated in Figure 13. The analysis reveals that, with the exception of HMCT, all other structures demonstrate a smooth and gradual folding mode. However, in comparison to the BMT structure, the inner and outer tubes of the BMCT structure exhibit a more pronounced shape difference, resulting in force dissipation not only during axial compression but also longitudinally. Consequently, all BMCT structures exhibit superior impact resistance compared to their BMT counterparts, with a notable increase in CFE of 41.04%. Figure 14 further depicts the force-displacement curves and energy absorption curves for each group of structures, indicating that the force-displacement curves for the BMCT structure are generally more stable than those of the BMT structure. Figure 15 presents a comparison of impact resistance indicators, which more clearly illustrates that all BMCT structures outperform their corresponding BMT structures in terms of impact resistance. Specific data can be found in Table 4.

## 5. Theoretical Analysis

From the aforementioned analysis, it can be seen that, compared with the traditional multi-cell tubes, the bio-inspired multi-cell concave tube (BMCT) proposed in this paper has significantly improved its impact resistance. To deeply reveal its energy absorption mechanism, in this section, based on the simplified super folding element (simplified super folding element, SSFE) theory [38,41], the theoretical expression of the average breaking force of BMCT is derived. According to the SSFE theory, assuming that all folding forms are consistent during the wrinkling deformation process, for any complete fold, according to the principle of energy conservation, the external force does most of the work to dissipate in the bending deformation energy and membrane deformation energy of BMCT, which can be expressed as [42]:

(1) $$2HkMCF=W_{bending}+W_{membrance}$$

In this context, 2HkMCF denotes the work executed by external forces, 2H represents the wavelengths of two folds, W_bending_ signifies the energy dissipated via bending deformation, and W_membrane_ refers to the energy dissipated through membrane deformation. It is important to note that no folded structure can be entirely flattened during the folding process. Therefore, an effective collapse distance coefficient (k) must be introduced to characterize this phenomenon, with its value typically ranging between 0.7 and 0.8 [43,44]. In this study, the value of k is established at 0.75.

### 5.1. Bending Deformation Energy

During the bending process of the sheet material, three fixed plastic hinges are usually formed, as shown in Figure 16. The energy generated by the bending deformation is mainly dissipated through the rotational process of these three hinges, and the energy expression is as follows:

(2) $$W_{bending}=\sum_{i=1}^{3}\theta_{i}M_{0}L_{c}$$

The rotation angles of the three shaping hinge lines, *θ_i_*, are π/2, π/2, and π, respectively. Thus, the bending energy can be expressed as follows:

(3) $$W_{bending}=2\pi M_{0}L_{c}$$

Here, *L*c represents the length of the plate, and M_0_ denotes the fully plastic bending moment, and its calculation formula is $M_{0}=\frac{\sigma_{0}t^{2}}{4}$. The flow stress is $\sigma_{0}$, defined in terms of $\sigma_{0}=\sqrt{\frac{\sigma_{y}\sigma_{u}}{1+n}}$, the yield stress of the material, $\sigma_{y}$, the ultimate stress of the material, $\sigma_{u}$, and n, the hardening index of the material. In this section, the value of n is set at 0.06.

### 5.2. Film Deformation Energy

**Figure 17:**
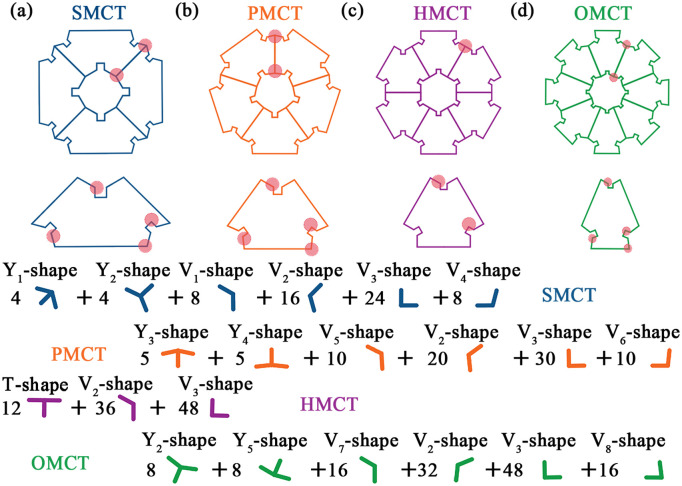
The constituent units of various BMCT structures.(**a**) SMCT; (**b**) PMCT; (**c**) HMCT; (**d**) OMCT.

The energy dissipated by the membrane exhibits variability across different units. Consequently, the total membrane energy dissipation must be ascertained by calculating the energy contribution of each fundamental constituent unit. As illustrated in Figure 17, the constituent units of each order of BMCT can be categorized into two types: two-panel units and three-panel units. The two-panel units are denoted as V1, V2, V3, V4, V5, V6, V7, and V8 (V-panel), while the three-panel units consist of Y1 shape, Y2 shape, Y3 shape, Y4 shape, Y5 shape (Y-shape), and T shape (T-shape).

In the symmetrical panel mode, the three-panel units are further subdivided into two forms. The first form occurs when the angle formed by two adjacent panels is equal to 180°, exemplified by the T-shape unit discussed in this paper. Conversely, the second form is characterized by an angle between two adjacent panels that does not equal 180°, as is the case with the Y-shape unit discussed herein.

In the symmetrical mode, the formula for the membrane deformation energy dissipation of the one-panel unit [45] is presented in Formula (4):

(4) $$E_{m,sym}^{1-panel}=\frac{{4M}_{0}H^{2}}{t}$$

The formula for the energy dissipation associated with film deformation in the two-panel unit [46] is delineated in Formula (5):

(5) $$E_{m,sym}^{2-panel}=\frac{{4M}_{0}H^{2}\tan\theta }{t(\tan\theta +0.05/\tan\theta )/1.1}$$

The V1-shape unit, classified as a two-panel unit, has a corresponding angle θ of 120.35°. Substituting the above values into Formula (5), the expression for the film deformation energy of the V1-shape unit can be derived, as indicated in Formula (6):

(6) $$E_{m,sym}^{V1-shape}=\frac{{4.33M}_{0}H^{2}}{t}$$

The V2-shape unit also belongs to the two-panel category, with a corresponding angle θ of 120°. Substituting the above values into Formula (5), the expression for the film deformation energy of the V2-shape unit can be obtained, as shown in Formula (7):

(7) $$E_{m,sym}^{V2-shape}=\frac{{4.33M}_{0}H^{2}}{t}$$

The V3-shape unit, also a two-panel unit, corresponds to an angle θ of 90°. Substituting this value into Formula (5) yields the expression for the film deformation energy of the V3-shape unit, as presented in Formula (8):

(8) $$E_{m,sym}^{V3-shape}=\frac{{4.4M}_{0}H^{2}}{t}$$

The V4-shape unit is characterized as a two-panel unit with a corresponding angle θ of 105°. Substituting the above values into Formula (5), the expression for the film deformation energy of the V4-shape unit can be derived, as indicated in Formula (9):

(9) $$E_{m,sym}^{V4-shape}=\frac{{4.39M}_{0}H^{2}}{t}$$

The V5-shape unit, another two-panel unit, corresponds to an angle θ of 120.04°. Substituting this value into Formula (5) allows for the derivation of the expression for the film deformation energy of the V5-shape unit, as shown in Formula (10).

(10) $$E_{m,sym}^{V5-shape}=\frac{{4.33M}_{0}H^{2}}{t}$$

The V6-shape unit is categorized as a two-panel unit, with an associated angle θ of 96°. Substituting the above values into Formula (5), the expression for the film deformation energy of the V6-shape unit can be derived, as depicted in Formula (11):

(11) $$E_{m,sym}^{V6-shape}=\frac{{4.4M}_{0}H^{2}}{t}$$

The V7-shape unit, classified as a two-panel unit, has a corresponding angle θ of 120.3°. Substituting the above values into Formula (5), the expression for the film deformation energy of the V7-shape unit can be derived, as shown in Formula (12):

(12) $$E_{m,sym}^{V7-shape}=\frac{{4.33M}_{0}H^{2}}{t}$$

The V8-shape unit, a two-panel unit, is associated with an angle θ of 82.5°. Substituting the above values into Formula (5), the expression for the film deformation energy of the V8-shape unit can be derived, as indicated in Formula (13):

(13) $$E_{m,sym}^{V8-shape}=\frac{{4.4M}_{0}H^{2}}{t}$$

In the symmetrical panel mode, the film deformation energy of the first type of three-panel unit can be determined according to Formula (14) [47,48]:

(14) $$E_{m,sym}^{3-panel}=4M_{0}\frac{H^{2}}{t}(\frac{\tan\alpha }{(\tan\alpha +0.05/\tan\alpha )/1.1}+2\tan\frac{\alpha }{2})$$

The T-shaped unit represents a specific instance within the first category of three-panel units, with a corresponding angle θ of 90°. Substituting the above angle into Formula (14), the expression for the film deformation energy of the T-shaped unit can be derived, as shown in Formula (15):

(15) $$E_{m,sym}^{T-shape}=\frac{{12.4M}_{0}H^{2}}{t}$$

In the symmetrical panel mode, the film deformation energy of the second type of three-panel unit can be computed using Formula (16):

(16) $$E_{m,sym}^{Y-shape}={2M}_{0}\frac{H^{2}}{t}(4\tan\left(\frac{\theta }{4}\right)+2\sin\frac{\theta }{2}+3\sin\theta )$$

The Y1-shape unit is categorized as a second type of three-panel unit, with a corresponding angle θ of 60°. Substituting this angle into Formula (16) yields the expression for the film deformation energy of the Y1-shape unit, as presented in Formula (17):

(17) $$E_{m,sym}^{Y1-shape}=\frac{{9.34M}_{0}H^{2}}{t}$$

The Y2-shape unit, also part of the second type of three-panel unit, has a corresponding angle θ of 105°. Substituting this value into Formula (16) allows for the derivation of the expression for the film deformation energy of the Y2-shape unit, as delineated in Formula (18):

(18) $$E_{m,sym}^{Y2-shape}=\frac{{12.91M}_{0}H^{2}}{t}$$

The Y3-shape unit, also classified as a second type of three-panel unit, corresponds to an angle θ of 78°. Substituting the above values into Formula (16), the expression for the film deformation energy of the Y3-shape unit can be derived, as illustrated in Formula (19):

(19) $$E_{m,sym}^{Y3-shape}=\frac{{11.22M}_{0}H^{2}}{t}$$

The Y4-shape unit is classified as a second type of three-panel unit, with a corresponding angle θ of 96°. Substituting the above angle into Formula (16), the expression for the film deformation energy of the Y4-shape unit can be obtained, as shown in Formula (20):

(20) $$E_{m,sym}^{Y4-shape}=\frac{{12.5M}_{0}H^{2}}{t}$$

The Y5-shape unit, also categorized as a second type of three-panel unit, has a corresponding angle θ of 82.5°. Substituting the above values into Formula (16), the expression for the film deformation energy of the Y5-shape unit can be derived, as illustrated in Formula (21):

(21) $$E_{m,sym}^{Y5-shape}=\frac{{11.6M}_{0}H^{2}}{t}$$

Ultimately, the deformation energy contributions of the films for each fundamental unit are aggregated to yield the total film deformation energy W_membrane_ for various BMCT structures. The corresponding expression is delineated in Formula (22):

(22) $$\begin{array}{ll} W_{membrane} & ={n_{V1}E}_{m,sym}^{V1-shape}+{n_{V2}E}_{m,sym}^{V2-shape}+{n_{V3}E}_{m,sym}^{V3-shape}\\ & +{n_{V4}E}_{m,sym}^{V4-shape}+{n_{V5}E}_{m,sym}^{V5-shape}+{n_{V6}E}_{m,sym}^{V6-shape}\\ & +{n_{V7}E}_{m,sym}^{V7-shape}{n_{V8}E}_{m,sym}^{V8-shape}\\ & ++{n_{T}E_{T-shape}+n}_{Y1}E_{m,sym}^{Y1-shape}+n_{Y2}E_{m,asym}^{Y2-shape}\\ & +n_{Y3}E_{m,asym}^{Y3-shape+n_{Y2}E_{m,asym}^{Y2-shape}}+n_{Y4}E_{m,asym}^{Y4-shape}\\ & +n_{Y5}E_{m,asym}^{Y5-shape} \end{array}$$

The variables *n_V_*_1_, *n_V_*_2_, *n_V_*_3_, *n_V_*_4_, *n_V_*_5_, *n_V_*_6_, *n_V_*_7_, *n_V_*_8_, *n_T_*, *n_Y_*_1_, *n_Y_*_2_, *n_Y_*_3_, *n_Y_*_4_, and *n_Y_*_5_ denote the quantities of V1-shape, V2-shape, V3-shape, V4-shape, V5-shape, V6-shape, V7-shape, V8-shape, T-shape, Y1-shape, Y2-shape, Y3-shape, Y4-shape, and Y5-shape, respectively. The quantities of the fundamental constituent units of various BMCT structures are presented in Table 5. Table 6 shows the contribution of each geometric unit to energy absorption under different structural deformations.

By integrating formulas (4) through (22), the total membrane deformation energy, denoted as W_membrane_, of the BMCT can be derived as indicated in Formula (23):

(23) $$\begin{array}{ll} W_{membrane} & =\frac{{4.33n_{V1}M}_{0}H^{2}}{t}+\frac{{4.33n_{V2}M}_{0}H^{2}}{t}+\frac{{4.4n_{V3}M}_{0}H^{2}}{t}\\ & +\frac{{4.39n_{V4}M}_{0}H^{2}}{t}+\frac{{4.33n_{V5}M}_{0}H^{2}}{t}+\frac{{4.4n_{V6}M}_{0}H^{2}}{t}\\ & +\frac{{4.33n_{V7}M}_{0}H^{2}}{t}+\frac{{4.4n_{V8}M}_{0}H^{2}}{t}+\frac{{12.4n_{T}M}_{0}H^{2}}{t}\\ & +\frac{{9.34n_{Y1}M}_{0}H^{2}}{t}+\frac{{12.91n_{Y2}M}_{0}H^{2}}{t}+\frac{{11.22n_{Y3}M}_{0}H^{2}}{t}\\ & +\frac{{12.5n_{Y4}M}_{0}H^{2}}{t}+\frac{{11.6n_{Y5}M}_{0}H^{2}}{t} \end{array}$$

### 5.3. Average Crushing Force

Substitute Formula (3) and (23) into Formula (1), and we obtain Formula (24):

(24) $$\begin{array}{ll} 2HkMCF=2\pi M_{0}L_{c} & +(4.33n_{V1}+4.33n_{V2}+4.4n_{V3}+4.39n_{V4}+4.33n_{V5}\\ & +4.4n_{V6}+4.33n_{V7}+4.4n_{V8}+12.4n_{T}+9.34n_{Y1}+12.91n_{Y2}\\ & +11.22n_{Y3}+12.5n_{Y4}+11.6n_{Y5})\frac{M_{0}H^{2}}{t} \end{array}$$

Then, divide both sides of Formula (24) by 2Hk simultaneously, and the calculation Formula for MCF (25) is obtained:

(25) $$\begin{array}{ll} MCF=\frac{\pi M_{0}L_{c}}{kH} & +(4.33n_{V1}+4.33n_{V2}+4.4n_{V3}+4.39n_{V4}+4.33n_{V5}+4.4n_{V6}\\ & +4.33n_{V7}+4.4n_{V8}+12.4n_{T}+9.34n_{Y1}+12.91n_{Y2}\\ & +11.22n_{Y3}+12.5n_{Y4}+11.6n_{Y5})/2)\frac{M_{0}H}{kt} \end{array}$$

Based on the quasi-static condition, $\frac{\partial MCF}{\partial H}=$ 0, Formula (26) can be obtained:

(26) $$H=\sqrt{\frac{\pi L_{c}t}{\left(\begin{array}{c} 4.33n_{V1}+4.33n_{V2}+4.4n_{V3}+4.39n_{V4}+4.33n_{V5}+\\ 4.4n_{V6}+4.33n_{V7}+4.4n_{V8}+12.4n_{T}+9.34n_{Y1}\\ +12.91n_{Y2}+11.22n_{Y3}+12.5n_{Y4}+11.6n_{Y5} \end{array}\right)/2}}$$

By substituting Formula (26) back into Formula (25), the calculation formula for the MCF of the BMCT without the half-wavelength H (Formula (27)) can be obtained:

(27) $$MCF=\frac{2M_{0}}{k}\sqrt{\frac{\left(\begin{array}{c} 2.165n_{V1}+2.165n_{V2}+2.2n_{V3}+2.195n_{V4}+2.165n_{V5}+\\ 2.2n_{V6}+2.165n_{V7}+2.2n_{V8}+6.2n_{T}+4.67n_{Y1}\\ +6.455n_{Y2}+5.61n_{Y3}+6.25n_{Y4}+5.8n_{Y5} \end{array}\right)\pi L_{c}}{t}}$$

By substituting $M_{0}=\frac{\sigma_{0}t^{2}}{4}$ into Formula (27), the simplified calculation formula for MCF (28) can be obtained:

(28) $$MCF=\frac{\sigma_{0}t}{2k}\sqrt{\left(\begin{array}{c} 2.165n_{V1}+2.165n_{V2}+2.2n_{V3}+2.195n_{V4}+2.165n_{V5}+\\ 2.2n_{V6}+2.165n_{V7}+2.2n_{V8}+6.2n_{T}+4.67n_{Y1}\\ +6.455n_{Y2}+5.61n_{Y3}+6.25n_{Y4}+5.8n_{Y5} \end{array}\right)\pi L_{c}t}$$

The mean crushing force (MCF) in the aforementioned formula was derived under quasi-static conditions, neglecting dynamic effects. However, under dynamic conditions, the energy absorption capacity of the structure is significantly enhanced due to inertia effects compared to quasi-static scenarios. Consequently, it is essential to introduce a dynamic enhancement factor $\lambda_{i}$, (i = S, P, H, O, the initial letters of the corresponding structural abbreviations), into the theoretical expression. This dynamic enhancement factor, as indicated by the research conducted by Langseth et al. [43,46], $\lambda_{i}$, ranges from 1.05 to 1.3. The study revealed that the MCF of various BMCT structures is predominantly influenced by the type and quantity of corner elements. In this research, the dynamic coefficient $\lambda_{i}$ for BMCT-S/P/H/O were designated as 1.215, 1.175, 1.133, and 1.253, respectively, to adjust the previously derived MCF and account for the inertia effect. Thus, the modified MCF_D_ expression under dynamic conditions, as presented in Formula (29), is provided:

(29) $${MCF}_{D}=\lambda_{i}MCF$$

To validate the reliability of the derived theoretical model, the theoretical predictions incorporating dynamic coefficients were compared with finite element simulation results. Table 7 presents a comparison between the theoretical predictions and finite element simulation results for various BMCT structures under conditions of uniform wall thickness. The maximum absolute average error between the theoretical predictions and finite element simulations was found to be merely 8.15%, indicating a strong correlation between the theoretical and numerical results. Therefore, this theoretical formula can reliably predict the MCF of various BMCT pipes, ensuring a high degree of accuracy.

Theoretical analysis suggests that the average deviation between the theoretical prediction values of various BMCT structures and the finite element simulation results does not exceed 8.15%. This finding presents a high-precision and cost-effective computational method for optimizing the crashworthiness of BMCT structures in future applications.

## 6. Conclusions

Drawing upon the principles of biomimicry and hierarchical design, a novel bio-inspired multi-cell concave tube has been proposed. Through numerical simulations, the impact resistance performance of the BMCT under axial compressive loads was examined, and a comprehensive analysis of its impact resistance performance was conducted. Concurrently, a theoretical model was established for further investigation. The results indicate that:

(1) Utilizing 3D printing technology, both the tensile material components and the models were fabricated. The disparity between the simulation and experimental results was merely 9.88%, thereby validating the reliability of the simulation experiment. Furthermore, it was demonstrated that wall thickness significantly influences the deformation mode of the BMCT.
(2) Comparative experiments involving various hierarchical structures under identical mass conditions were designed to assess the impact of differing hierarchies on the crashworthiness of the structures. An increase in hierarchy resulted in a maximum increase in specific energy absorption (SEA) of 41.78% and a maximum increase in crush force efficiency (CFE) of 21.22%.
(3) In comparison to traditional thin-walled tubes, the BMCT exhibits superior crashworthiness. The inner concave hierarchical design significantly enhances the crashworthiness of thin-walled tubes, with the maximum increase in CFE reaching 41.04%. However, unlike conventional multi-cell tubes, the impact resistance of the BMCT does not exhibit a monotonous increase with the number of cells; optimal wall thickness varies according to different structural deformations.
(4) A theoretical model based on the simplified super folding element theory was developed to predict the MCF of the BMCT. The numerical results were found to align closely with theoretical predictions.

Despite BMCT’s exceptional energy-absorption capabilities, its intricate structure presents manufacturing challenges. With the rapid advancement of metal 3D printing technology, large-scale production and cost management of such complex layered structures are anticipated to become feasible in the future, thereby facilitating their application in practical engineering domains.

## Figures and Tables

**Table 1:** Indicators of crashworthiness.

Indicator	Definition	Displayed Formulae
EA	The total energy absorbed by a thin-walled structure during effective compression	$EA=\int_{0}^{d}F(x)dx$
SEA	The energy absorbed per unit mass	$SEA=\frac{EA}{m}$
MCF	The ratio of the total energy absorbed to the impact distance	$MCF=\frac{EA}{d}$
CFE	The ratio of MCF to IPCF	$CFE=\frac{MCF}{IPCF}\times 100\%$

**Table 2:** Detailed data of mesh size analysis.

Mesh Size (mm)	EA (J)	Diff (%)	IPCF (kN)	Diff (%)
0.8	3613.04	-	74.56	-
1.0	3819.58	5.72	74.85	0.89
1.2	3689.56	2.12	75.14	1.28
1.5	3827.88	5.95	75.61	1.67
1.8	4001.13	10.74	75.87	2.31
2.0	4281.93	18.51	75.93	2.65

**Table 3:** Crashworthiness data with the same mass.

	t (mm)	m(g)	MCF (kN)	IPCF (kN)	SEA (J/g)	CFE (%)
SMCT	0.84	129.40	31.76	50.79	19.63	62.53
PMCT	0.84	129.40	34.28	52.73	21.20	65.01
HMCT	0.80	129.40	33.11	54.68	20.47	60.55
OMCT	0.71	129.40	44.69	58.96	27.63	75.80
SMCT	1.05	161.75	43.05	66.47	21.29	64.76
PMCT	1.05	161.75	46.09	69.55	22.80	66.27
HMCT	1.00	161.75	46.12	75.14	22.81	61.38
OMCT	0.89	161.75	61.03	78.22	30.19	78.03
SMCT	1.26	194.11	58.17	82.83	23.97	70.22
PMCT	1.25	194.11	60.51	87.18	24.94	69.41
HMCT	1.20	194.11	61.79	92.08	25.47	67.11
OMCT	1.06	194.11	73.77	97.81	30.41	75.42

**Table 4:** Crashworthiness data with other structures.

	t (mm)	m(g)	MCF (kN)	IPCF (kN)	SEA (J/g)	CFE (%)
SMCT	1.05	161.75	43.05	66.47	21.29	64.76
SMT	1.02	161.75	29.45	64.14	14.57	45.92
PMCT	1.05	161.75	46.09	69.55	22.80	66.27
PMT	1.07	161.75	32.66	64.18	16.15	50.89
HMCT	1.00	161.75	46.12	75.14	22.81	61.38
HMT	1.07	161.75	34.62	64.21	17.12	53.92
OMCT	0.89	161.75	61.03	78.22	30.19	78.03
OMT	1.02	161.75	39.70	64.80	19.64	61.27

**Table 5:** Specific parameters of the constituent units of various BMCT structures.

		1	2	3	4	5	6
SMCT	type	Y1-shape	Y2-shape	V1-shape	V2-shape	V3-shape	V4-shape
	angle/°	60/60/240	105/105/150	120.35	120	90	105
	numbers	4	4	8	16	24	8
PMCT	type	Y3-shape	Y4-shape	V5-shape	V2-shape	V3-shape	V6-shape
	angle/°	78/78/204	96/96/168	120.04	120	90	96
	numbers	5	5	10	20	30	10
HMCT	type	T-shape	V2-shape	V3-shape	-	-	-
	angle/°	90/90/180	120	90	-	-	-
	numbers	12	36	48	-	-	
OMCT	type	Y2-shape	Y5-shape	V7-shape	V2-shape	V3-shape	V8-shape
	angle/°	105/105/150	82.5/82.5/195	120.3	120	90	82.5
	numbers	8	8	16	32	48	16

**Table 6:** Contribution of each geometric unit to energy absorption.

		1	2	3	4	5	6
SMCT	Type (-shape)	4Y1	4Y2	8V1	16V2	24V3	8V4
	Proportion (%)	11.20	15.48	10.38	20.76	31.65	10.53
PMCT	Type (-shape)	5Y3	5Y4	10V5	20V2	30V3	10V6
	Proportion (%)	13.22	14.72	10.20	20.40	31.10	10.37
HMCT	Type (-shape)	12T	36V2	48V3	-	-	-
	Proportion (%)	28.84	30.22	40.94	-	-	
OMCT	Type (-shape)	8Y2	8Y5	16V7	32V2	48V3	16V8
	Proportion (%)	16.46	5.52	11.04	22.09	33.66	11.22

**Table 7:** Error between the theoretical predictions of MCF for various BMCT tubes and the finite element simulation results.

Tubes	t (mm)	λ	*L*c (mm)	Theo. (kN)	FE. (kN)	Diff. (%)	Mean Diff. (%)
SMCT	0.8	1.215	572.12	29.69	31.05	−4.38	3.44
SMCT	1.2	1.215	572.12	54.54	55.26	−1.30	
SMCT	1.6	1.215	572.12	83.97	80.04	4.91	
SMCT	2.0	1.215	572.12	117.35	113.74	3.17	
PMCT	0.8	1.175	572.90	30.65	33.47	−8.43	4.54
PMCT	1.2	1.175	572.90	59.54	56.82	4.79	
PMCT	1.6	1.175	572.90	91.66	88.83	3.19	
PMCT	2.0	1.175	572.90	128.1	125.88	1.76	
HMCT	0.8	1.133	599.10	35.23	33.11	6.40	5.28
HMCT	1.2	1.133	599.10	64.72	61.79	4.74	
HMCT	1.6	1.133	599.10	99.65	106.36	−6.31	
HMCT	2.0	1.133	599.10	139.25	144.57	−3.68	
OMCT	0.8	1.253	676.48	47.69	52.74	−9.58	8.15
OMCT	1.2	1.253	676.48	87.62	92.20	−4.97	
OMCT	1.6	1.253	676.48	134.89	132.93	1.47	
OMCT	2.0	1.253	676.48	188.52	161.69	16.59	

## Data Availability

The original contributions presented in this study are included in the article. Further inquiries can be directed to the corresponding author.
